# Intergenerational effects of maternal birth season on offspring size in rural Gambia

**DOI:** 10.1098/rspb.2012.1363

**Published:** 2012-08-15

**Authors:** Ian J. Rickard, Alexandre Courtiol, Andrew M. Prentice, Anthony J. C. Fulford, Tim H. Clutton-Brock, Virpi Lummaa

**Affiliations:** 1 Department of Animal and Plant Sciences, University of Sheffield, Sheffield S10 2TN, UK; 2 Wissenschaftskolleg zu Berlin, Berlin, Germany; 3 Department of Evolutionary Genetics, Leibniz Institute for Zoo and Wildlife Research, Berlin, Germany; 4 MRC Keneba, Medical Research Council Laboratories, Banjul, The Gambia; 5 MRC International Nutrition Group, London School of Hygiene and Tropical Medicine, London WC1E 7HT, UK; 6 Large Animal Research Group, Department of Zoology, University of Cambridge, Cambridge CB2 3EJ, UK

**Keywords:** intergenerational effects, maternal effects, birth season, epigenetics, maternal constraint, humans

## Abstract

Environmental conditions experienced in early life can influence an individual's growth and long-term health, and potentially also that of their offspring. However, such developmental effects on intergenerational outcomes have rarely been studied. Here we investigate intergenerational effects of early environment in humans using survey- and clinic-based data from rural Gambia, a population experiencing substantial seasonal stress that influences foetal growth and has long-term effects on first-generation survival. Using Fourier regression to model seasonality, we test whether (i) parental birth season has intergenerational consequences for offspring *in utero* growth (1982 neonates, born 1976–2009) and (ii) whether such effects have been reduced by improvements to population health in recent decades. Contrary to our predictions, we show effects of maternal birth season on offspring birth weight and head circumference only in recent maternal cohorts born after 1975. Offspring birth weight varied according to maternal birth season from 2.85 to 3.03 kg among women born during 1975–1984 and from 2.84 to 3.41 kg among those born after 1984, but the seasonality effect reversed between these cohorts. These results were not mediated by differences in maternal age or parity. Equivalent patterns were observed for offspring head circumference (statistically significant) and length (not significant), but not for ponderal index. No relationships were found between paternal birth season and offspring neonatal anthropometrics. Our results indicate that even in rural populations living under conditions of relative affluence, brief variation in environmental conditions during maternal early life may exert long-term intergenerational effects on offspring.

## 1. Introduction

Brief periods of environmental adversity experienced in very early development may not only have permanent consequences for the health of the individual [1] but also that of their offspring [2,3]. Intergenerational effects of the parental environment on offspring growth, development and health may occur through mechanisms such as maternal physical or physiological constraint [4] or through maternal and paternal epigenetic inheritance [2]. Such phenomena, while currently poorly understood, have major implications for epidemiology and public health not only because they represent a means by which the effects of environmental risk factors may perpetuate for decades, but also because they may suggest opportunities for intervention. The evidence for intergenerational effects of the early environment is strongest in experimental studies of animal models of human disease [2,3]. These studies show that exposing rats to pre- or early postnatal dietary restriction or stress results in reduced foetal growth of their offspring [5–7].

Few studies have so far been able to investigate intergenerational effects of the parental early environment in humans. Maternal birth weight has been found, inter alia, to be negatively related to offspring adulthood blood pressure [8,9] and metabolic disease risk [8,10,11]. Since maternal birth weight is influenced by a variety of factors, including genes, addressing the possibility of early environmental effects on individuals and their offspring is best addressed by ‘natural experimental’ conditions (variation in the external environment itself). However, opportunities are rare. Several studies show that maternal environmental conditions or socio-economic circumstances in adults have intergenerational effects on birth metrics [10–12], and a growing number of studies have addressed the role of maternal childhood environment on offspring birth outcomes [13–16], but the specific role of the maternal pre- or early postnatal environment in these cases is unclear.

Effects of maternal early environment on offspring growth have been studied most extensively in the Dutch ‘hunger winter’ cohorts. Lumey and co-workers [17–20] studied the effects of maternal prenatal exposure to a famine that took place in the Netherlands during World War II on next generation prenatal growth. They found that the normal parity-related increase in birth weight from first- to second-born offspring was absent in the offspring of women exposed to famine early in their own gestation [17,19,20]. After controlling for maternal birth weight, which exhibited trimester of exposure-specific effects, there was no modifying effect of maternal famine exposure on the parity progression in offspring birth weight [18]. Painter *et al*. [21] studied the same population and found that independently of parity, neonates whose mothers had at some point been prenatally exposed to the famine were equivalent in weight, but shorter (and consequently had a higher relative weight—‘ponderal index’) than controls. Various distinctions between the methodologies of the studies by Lumey and co-workers and that of Painter *et al.* could account for these differences. Painter *et al.* [21] also found some suggestion of offspring adult health being compromised by maternal *in utero* famine exposure.

A few studies of populations other than the Dutch hunger winter cohort examine effects of maternal early environment on offspring growth, and the results are again inconsistent. One paper examining intergenerational effects on offspring growth showed that the offspring of women born during the Chinese ‘Great Leap Forward’ Famine of 1959–1961 had lower height- and weight-for-age [22]. A recent study of intergenerational effects of maternal *in utero* exposure to Ramadan fasting looked separately at effects of exposure during each of the trimesters of pregnancy on four birth outcomes in offspring of both sexes [23]. The only statistically significant intergenerational effect uncovered was a positive effect of second trimester exposure on birth length in boys. However, statistical power to detect biologically meaningful effects is lost by the division of exposed mothers into trimester-specific cohorts, a problem not shared by alternative procedures that take into account temporal continuity, see below. Additionally, although Ramadan causes perturbations in the patterns of eating, with possible implications in pregnancy due to the phenomenon of ‘accelerated starvation’ [24], it does not necessarily result in a restriction in overall nutrient intake.

Because of the mothers' role in providing an environment and provisioning for offspring from conception until weaning, much of the evidence for intergenerational effects of the early environment on offspring has come through looking at apparent effects of the maternal early environment. However, a mechanism that is being increasingly seen as playing an important role in mediating effects of the early environment on phenotype, and which could operate through both fathers and mothers, is epigenetic programming effects (e.g. methylation patterns), by which gene regulation is permanently altered during early life [25,26]. During early embryonic development, gene expression is programmed, in a largely tissue-specific pattern, by DNA methylation [25]. Maternal and paternal condition, diet and other environmental factors have been shown, experimentally, to modify these patterns, with results that can endure for several generations [25]. Recent evidence from the Dutch Hunger winter cohorts [27,28] as well as rural Gambia [29] have implicated the early environment in modification of methylation status of several genes, including those important for growth and metabolism [28] and ‘metastable epialleles’—epigenetic patterns that are invariant across cell type. While in the above case, the critical period is likely to be shortly after gametogenesis, a window for environmental influence during spermatogenesis has been suggested to account for observations relating individuals' health and longevity to the environment experienced by their fathers between the ages of eight and 12 years [30,31].

Thus, the evidence from humans offers some support to the findings from studies of animal models that parental exposure to adverse environmental conditions during critical periods of development can have consequences for offspring phenotype. Unfortunately, few datasets combine measures of parental early environment with offspring outcomes. Changes in environmental conditions as a result of birth season provide one possibility for identifying and comparing groups of individuals experiencing different environmental conditions in early life. In particular, long-term data from traditional societies in which seasonal patterns of farm work, hunger and disease exposure have a significant impact on individual health, and nutritional status may prove more useful in understanding the interactions between the developing human and the environment [32–34]. Such settings are also relevant to the conditions under which a substantial portion of humans currently live.

In this study, we investigate relationships between maternal and paternal early conditions and offspring neonatal anthropometrics in rural Gambia. Since 1950, residents of several villages in the West Kiang district of the country have been studied and treated by the UK Medical Research Council. Seasonal variation in food supply culminates in an acute shortage of energy when agricultural workload is highest; termed the ‘hungry season’. The consequent energy shortage, compounded by increased exposure to infectious diseases, such as malaria, leads to a marked seasonal reduction in the weights of pregnant and lactating women [35], and has traditionally been accompanied by a higher overall mortality rate [36]. Babies born between July and December are exposed to these stressors during late gestation when intrauterine growth rate is highest, and as a consequence they are generally lighter than babies born between January and June [35,37]. Furthermore, individuals born between July and December have a 10-fold increased risk of premature adulthood death [33], primarily owing to infectious disease [38], indicating that birth season can have delayed but profound effects on individual phenotype.

We test two specific predictions: First, we predict that parental birth season will have long-term consequences that impact offspring growth. Second, we predict that changes in the provisioning of medical care over recent years over which the population has been monitored [1,39] will have caused these seasonal changes to decline relative to earlier years, or to disappear.

## 2. Material and methods

Since 1950, residents of three villages in the West Kiang district of The Gambia (Keneba, Kantong Kunda and Manduar) have been studied and treated by the UK Medical Research Council. This arrangement was initiated by Ian McGregor, when the population of the largest village, Keneba, numbered approximately 700 inhabitants, mostly of Mandinka ethnicity. Traditionally, the population has subsisted largely on crops of rice, sorghum and millet, with a single cash crop of groundnuts. Over the study period, there has been incremental improvement in the provision of the healthcare to the local population, most notably in the form of increased antenatal and natal care since the 1970s. In 1974, a clinic was established to provide free medical care to residents of the three villages and the surrounding area, and to monitor the health of neonates, infants and children. Overall, under-five mortality rate declined from more than 40 per cent prior to 1970 to less than 10 per cent in the present day [2,3,39]. Fertility has also historically been high, with women giving birth to a total of around seven children on average [4,40]. The society is highly polygynous, and women spend virtually all of their adult lives married, beginning reproduction at about 18 years of age.

We analysed the effects of first maternal, and then paternal, birth season on the neonatal anthropometrics (birth weight, length, ponderal index and head circumference) of a total of 1982 singleton offspring born to 581 mothers (maternal cohort before 1975: *N* = 327 mothers, *N* = 1442 offspring; 1975–1984: *N* = 205 mothers, *N* = 483 offspring; after 1984; *N* = 49 mothers, *N* = 57 offspring) and on 942 singleton offspring born to 240 fathers (paternal cohort before 1975: *N* = 194 fathers, *N* = 862 offspring; 1975–1984: *N* = 48 fathers, *N* = 79 offspring). Tables 1 and 2 show the means, standard errors and sample sizes for each of these offspring birth metrics split by maternal and paternal birth season. Ponderal index, a measure of leanness, is calculated as weight divided by length cubed (weight/length^3^). Not all measures were available for all offspring. Mothers were born between 1950 and 1994, fathers between 1950 and 1984 and offspring between 1976 and 2009. Maternal age at offspring birth ranged from 14 to 47, and paternal age between 12 and 61. Offspring were measured within 24 h of birth by trained staff.

**Table 1.** Mean ± s.e. and sample sizes of birth metric, split by maternal birth cohort.

maternal cohort	weight (kg)	height (mm)	head circumference (mm)	ponderal index (kg m^–3^)
before 1975	2.96 ± 0.42 (*n* = 1413)	494.76 ± 25.03 (*n* = 687)	339.62 ± 13.31 (*n* = 1146)	24.55 ± 3.76 (*n* = 667)
1975–1984	2.92 ± 0.38 (*n* = 470)	491.16 ± 29.61 (*n* = 372)	341.80 ± 14.60 (*n* = 362)	25.17 ± 6.77 (*n* = 359)
after 1984	2.95 ± 0.38 (*n* = 56)	494.98 ± 24.63 (*n* = 40)	340.86 ± 14.25 (*n* = 37)	24.55 ± 3.45 (*n* = 39)
total	2.95 ± 0.41 (*n* = 1939)	493.55 ± 26.69 (*n* = 1099)	340.16 ± 13.67 (*n* = 1545)	24.76 ± 4.98 (*n* = 1065)

**Table 2.** Mean ± s.e. and sample sizes of birth metric, split by paternal birth cohort.

paternal cohort	weight (kg)	height (cm)	head circumference (cm)	ponderal index (kg m^−3^)
before 1975	2.93 ± 0.40 (*n* = 837)	49.21 ± 2.55 (*n* = 570)	34.04 ± 1.40 (*n* = 690)	24.69 ± 4.68 (*n* = 555)
1975–1984	2.90 ± 0.38 (*n* = 76)	49.59 ± 2.19 (*n* = 67)	34.07 ± 1.34 (*n* = 67)	23.77 ± 2.92 (*n* = 64)
total	2.93 ± 0.40 (*n* = 913)	49.25 ± 2.52 (*n* = 637)	34.05 ± 1.39 (*n* = 757)	24.59 ± 4.54 (*n* = 619)

Birth weight split by maternal/paternal birth cohort and two-factor maternal birth season (January–January versus July–December) is shown in the electronic supplementary material, tables S1 and S2. In analysis, season of birth was modelled using Fourier series [2,37,41,42]. Fourier-based approaches allow the decomposition of any periodic function into a linear combination of simple oscillating functions (sines and cosines) parametrized by coefficients (the Fourier coefficients). Use of these terms come from trigonometric algebra used to express periodicity using the framework of the unit circle, and can potentially be used to fit very complex period signals (e.g. silhouettes of human body [43]). It is therefore possible to model continuously seasonality variation on any trait by fitting the Fourier coefficients of sine and cosine terms using the general framework of linear models. In particular, we modelled the seasonal component of the linear predictor as follows:
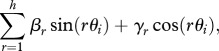
where *h* is the number of pairs of Fourier terms considered (the harmonic number); *β_r_* is the Fourier coefficient of the sine term of the *r* harmonic; *γ_r_* is the Fourier coefficient of the cosine term of the *r* harmonic and *θ_i_* is the point in the annual cycle when the individual *i* is born expressed in radians (e.g. 1 January ∼ 0; 1 July ∼ *π*; 31 December ∼ 2*π*).

For each of the four offspring birth anthropometrics, we produced a linear model that included as predictors the maternal/paternal and offspring birth season Fourier terms with a harmonic number *h =* 1 (offspring sine, offspring cosine, maternal sine and maternal cosine). This model also included as predictors maternal or paternal village (three levels); maternal cohort (before 1975; 1975–1984; after 1984); maternal age (linear and quadratic); paternal cohort (before 1975; 1975–1984, paternal analysis only); paternal age (linear and quadratic, paternal analysis only); maternal parity (two levels: parous and nulliparous) and offspring sex. Bearing in mind the number of interaction terms being considered (see below), in order to avoid producing highly parametrized models, we kept the number of factor levels to a minimum (e.g. two in the case of parity). For maternal/paternal birth cohort, we based our decision on the appropriate number of factor levels on previous research showing substantial difference in mortality rates between the periods as described earlier [2,3,39]. Expanding the number of factor levels (allowing a higher resolution of birth cohort) did not at any point improve model fit, see §3. Only one paternity was assigned to a male born after 1984, and so only the first two cohorts were considered in analysis of effects of paternal birth season. In addition to the fixed effects, we also considered maternal or paternal ID as a random effect to account for lack of independence between siblings. The linear mixed-effect models were fitted using the function lmer (package: lme4) from the R environment [44]. Likelihood ratio test comparing the fit to data of models with and without a given term indicated that term's statistical significance.

In addition to the first-order maternal Fourier terms, we included interactions between maternal/paternal Fourier terms and maternal parity, maternal cohort and maternal/paternal age (linear). This was because (i) effects of maternal early environment on offspring *in utero* growth have previously been found to be dependent on maternal parity [5–7,17–20], which is confounded with both maternal age and maternal cohort; (ii) maternal/paternal age may modify effects of environment on offspring [8,9,45]; (iii) it has been shown that birth season effects on mortality in this population have declined in recent years [8,39], so we predicted that the effects of maternal birth season will change over time. The interactions between paternal birth season terms and maternal birth cohort, maternal parity and maternal age were considered in the paternal analysis. Full details of all terms considered can be found in electronic supplementary material, tables S3–S10. Owing to the sex-specific nature of epigenetic programming effects, interactions between parental birth season terms and offspring sex were initially tested, but were removed from final models because in no cases was there an improvement to model fit as a result of doing this (all *p* > 0.21).

We determined that model fits were not improved either by increasing the harmonic numbers of maternal/paternal and offspring birth Fourier series (all *p* > 0.53 for comparisons of *h* = 1 versus 2), so seasonality is parsimoniously described by a continuous variation between two main different seasons per year, not more. We also produced alternative models to constrain fixed effects interacting with the maternal/paternal Fourier terms to modify the amplitude of seasonality [10–12,42]. To do this, we modelled interactions between both Fourier coefficients of a given harmonic and a given fixed effect by a single parameter common to both sine and cosine terms using the function nlme (package: nlme). None of the constrained models produced a better fit (baseline models initially fitted with the function lmer refitted using the function lme from package nlme to allow model comparison). Where maternal/paternal Fourier terms were significant (see §3), the general improvement in fit gained by using unconstrained models indicated the value of the additional parameters necessary for shifts in the timing (phase) of modes of the seasonal periodicity (in addition to the amplitude).

For each offspring neonatal anthropometric, in order to test the overall effect of mother seasonality, we compared the baseline model containing interactions between maternal/paternal birth season and first-order maternal/paternal birth season Fourier terms with one in which these effects were removed. Finally, we added several additional terms to each model that could help identify potential mechanisms by which maternal early environment could influence offspring growth, and determined whether their inclusion in the models mediated the maternal birth season effects. These were maternal adult height, maternal first recorded weight in infancy (if recorded before three months, adjusted linearly for age) and offspring gestational age (assessed within 5 days of birth by the Dubowitz method [13–16,46]). Data were incomplete for these mediating factors, so they were coded as three-level categories (approximate tertiles) with an additional level for missing data.

## 3. Results

### (a) Birth weight

**Figure 1.**
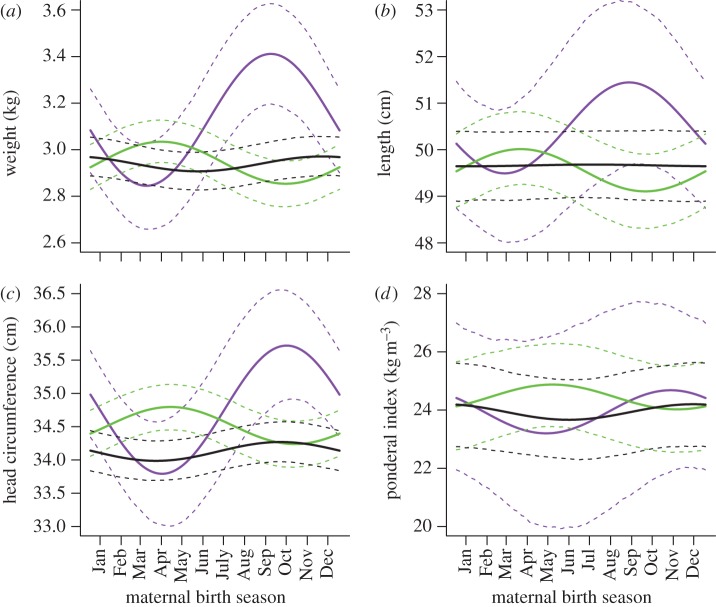
Cohort-specific effects of maternal birth season on offspring neonatal (*a*) weight, (*b*) length, (*c*) head circumference and (*d*) ponderal index. Lines represent predicted values for an average firstborn male born mid-year to a mother of median age from the village of Keneba. Mothers were born before 1975 (black solid line), 1975–1984 (green solid line) or after 1984 (purple solid line). Dashed lines represent corresponding boundaries of 95% CI (based on 5000 parametric bootstraps).

We analysed birth weights (mean ± s.d. = 2.95 ± 0.42 kg) of 1939 babies born to 576 mothers (range, 1–11; median, two offspring per mother). Comparison between models that did and did not contain maternal birth season terms showed that the better fit was obtained when maternal birth season terms were included (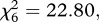 *p* = 0.0009, see the electronic supplementary material, table S3). This indicated that maternal birth season was related to offspring birth weight, but since the suite of maternal birth season terms included in the model comprised interactions between maternal age, maternal parity and maternal cohort as well as the first-order effect of maternal birth season, we removed each term separately and assessed changes to model fits in order to assess which of these were significant. The effect of maternal birth season was only revealed when the interaction between maternal birth season and maternal birth cohort (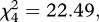 *p* < 0.0002) was retained (figure 1*a*), indicating that the effects of maternal birth season changed depending on the year in which a woman was born. Subsequently, we compared models with merged birth cohorts to our base model in order to determine whether different birth cohorts could be pooled without significantly impeding our model fit. The best model fit was obtained when all three cohort levels were retained (all *p* < 0.036 when compared with models in which any cohorts were merged), demonstrating that the relationship between maternal birth season and offspring birth weight was significantly different among all three maternal cohorts. Assessing the significance of maternal birth season within each cohort revealed no effect in the before-1975 cohort (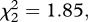 *p* = 0.40) but effects in both the 1975–1984 (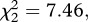 *p* = 0.024) and after-1984 (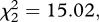 *p* = 0.0005) cohort. Although the power to detect an effect of maternal birth season differs between cohorts, the trend suggested by the *p*-values matches the one given by predicted differences in birth weight through the year. We observed almost no difference in the predicted birth weight before 1975 (figure 1), while in the 1975–1984 cohort, a peak of 3.03 kg and a trough of 2.85 kg were predicted for 15 October and 14 April, respectively, and for the after-1984 cohort, a peak of 3.41 kg and a trough of 2.84 kg were predicted for 23 March and 23 September. Maternal birth seasonality remained significant even after including in the models the potential mediators of maternal infancy weight, maternal adult height and offspring gestational age (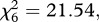 *p* = 0.0015), indicating that neither maternal size, gestation length nor the combination of these factors accounted for the results obtained (see the electronic supplementary material, figure S2*a*).

We analysed birth weights (mean ± s.d. = 2.94 ± 0.46 kg) of 913 babies born to 236 fathers (range, 1–23; median, two offspring per father). Model comparison showed that, overall, inclusion of paternal birth season terms did not improve model fit (
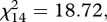
 *p* = 0.17, electronic supplementary material, table S4). There was no interaction between paternal birth season and paternal birth cohort (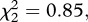 *p* = 0.65, see the electronic supplementary material, figure S1*a*) and no first-order effect of paternal birth season (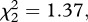 *p* = 0.50, compare electronic supplementary material, figure S1*a* with figure 1*a*).

### (b) Birth length

We analysed birth lengths (mean ± s.d. = 49.36 ± 2.67 cm) of 1099 babies born to 450 mothers (range, 1–9; median, two offspring per mother). Exclusion of maternal birth season terms did not reduce model fit (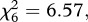 *p* = 0.36), indicating that maternal birth season did not significantly influence offspring birth length (see the electronic supplementary material, table S5). There were no improvements to model fit as a result of specific inclusion of interactions of maternal birth season with maternal cohort (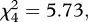 *p* = 0.22), or the first-order effect of maternal birth season after removal of the interaction (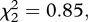 *p* = 0.65). However, the non-significant pattern of maternal seasonality in each of the respective cohorts is similar to that found for birth weight (figure 1*b*).

We analysed birth lengths (mean ± s.d. = 49.27 ± 30.20 kg) of 637 babies born to 195 fathers (range, 1–18; median, two offspring per father). Model comparison showed that overall, inclusion of paternal birth season terms did not improve model fit (
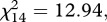
 *p* = 0.53, see the electronic supplementary material, table S6). There was no interaction between paternal birth season and paternal birth cohort (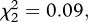 *p* = 0.96, electronic supplementary material, figure S1*b*) and no first-order effect of paternal birth season (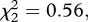 *p* = 0.75).

### (c) Birth head circumference

We analysed birth head circumferences (mean ± s.d. = 34.02 ± 2.54 cm) of 1545 babies born to 512 mothers (range, 1–10; median, two offspring per mother). Exclusion of all maternal birth season terms reduced model fit overall (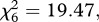 *p* = 0.0034, see the electronic supplementary material, table S7). As was the case with birth weight, maternal birth season interacted with maternal birth cohort (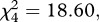 *p* = 0.0009 (figure 1*c*) and *post hoc* analysis revealed that the best model fit was obtained when all three cohort levels were retained (all *p* < 0.0044). Assessing the significance of maternal birth season in each cohort revealed no effect in the before-1975 cohort (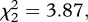 *p* = 0.14) but effects in the 1975–1984 cohort (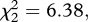 *p* = 0.041) and the after-1984 cohort (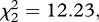 *p* = 0.0022). In the 1975–1984 cohort, a peak of 34.80 cm and a trough of 34.24 cm were predicted for 29 April and 26 October, respectively, while in the after-1984 cohort a peak of 35.72 cm and a trough of 33.79 cm were predicted for 15 October and 14 April. Maternal birth seasonality remained significant even after including in the models the potential mediators maternal infancy weight, maternal adult height and offspring gestational age (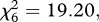 *p* = 0.0038) indicating that neither maternal size, gestation length nor the combination of these factors accounted for the maternal birth season patterns (see the electronic supplementary material, figure S2*c*).

We analysed birth head circumferences (mean ± s.d. = 34.16 ± 16.46 cm) of 757 babies born to 212 fathers (range, 1–18; median, two offspring per father). Model comparison showed that overall, inclusion of paternal birth season terms did not improve model fit (
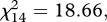
 *p* = 0.18, electronic supplementary material, table S8). There was no significant interaction between paternal birth season and paternal birth cohort (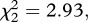 *p* = 0.23, see the electronic supplementary material, figure S1*c*) and no first-order effect of paternal birth season (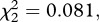 *p* = 0.96).

### (d) Birth ponderal index

We analysed birth ponderal index (weight/length^3^, mean ± s.d. = 2.48 ± 0.50) of 1065 babies born to 445 mothers (range, 1–9; median, two offspring per mother). Exclusion of maternal birth season terms did not reduce model fit (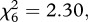 *p* = 0.89), indicating that maternal birth season terms overall did not modify offspring ponderal index (see the electronic supplementary material, table S9). As was the case with birth length, there were no improvements to model fit as a result of the interaction of maternal birth season with maternal cohort (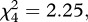 *p* = 0.69), or the first-order effect of maternal birth season (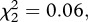 *p* = 0.97). The non-significant seasonal pattern for each of the three cohorts is shown in figure 1*d*.

We analysed birth ponderal index (weight/length^3^, mean ± s.d. = 24.83 ± 5.37) of 619 babies born to 193 fathers (range, 1–18; median, two offspring per father). Model comparison showed that overall, inclusion of paternal birth season terms did not improve model fit (
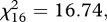
 *p* = 0.27, see the electronic supplementary material, table S10). Specific inclusion of the interaction between paternal birth season and paternal birth cohort did not significantly improve model fit (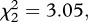 *p* = 0.22, see the electronic supplementary material, figure S1*d*) and there was no first-order effect of paternal birth season (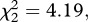 *p* = 0.12).

## 4. Discussion

An individual's experiences during their development can have consequences not only for their own phenotype, but also that of their offspring [2,3,17–20]. Such intergenerational effects are poorly understood in humans. We studied the relationships between maternal and paternal birth season and offspring neonatal anthropometrics (birth weight, length, head circumference and ponderal index) in rural Gambia, and found that the timing of maternal birth in relation to seasonal stressors predicted offspring birth weight and head circumference. Controlling for moderating effects of maternal age and parity, we did not find intergenerational effects of the maternal birth season among the earliest maternal cohorts, but such effects were apparent in those born since 1975, after which healthcare interventions had significantly reduced child mortality rates [39]. We must therefore reject the hypothesis that effects of maternal early environment become weaker or disappear in those recent years where health provisioning in the local population has improved. In addition, the reversal of the seasonal pattern between the two cohorts in which maternal birth season affected offspring growth defies simple explanation. These results are particularly heterogeneous, spanning from no effect of birth season, to two seasonal patterns that are phase-shifted by 180°.

To our knowledge, our study is the first in humans to examine simultaneously both maternal and paternal putative transgenerational effects of early life environment on offspring outcomes. We did not find any significant relationship between paternal birth season and offspring outcomes. However, because of differences in the distributions of paternal versus maternal birth years, we cannot determine whether or not the birth season effects might be apparent in the offspring of men truly contemporaneous to those women whose offspring displayed an intergenerational effect of maternal birth year. Paternity data are less complete than maternity data, and data are available on fewer offspring sired by men born since 1975 than born to women born over the same period. Some of the coefficients for the interaction between paternal birth season and paternal birth cohort are comparable to significant terms that correspond to mothers (e.g. coefficients for the ‘sine: born 75–84’ interaction coefficient for head circumference: 0.35 ± 0.14, *p* = 0.011 (mothers), 0.43 ± 0.31, *p* = 0.17 (fathers), see electronic supplementary material, tables S7 and S8). It is therefore entirely possible that comparable effects of paternal birth season do exist in recent cohorts, but insufficient data currently exist to detect this.

The observation that the environment experienced by women during their own early life can affect the intrauterine growth of their offspring adds to the findings from studies of long-term effects of early life exposure the Dutch hunger winter on offspring anthropometrics [17,18,18–21], and in other populations [21–23] as well as other studies showing within-generation effects of the early environment on phenotype in this Gambian population [29,33,37,38,47]. By exploiting the ‘natural experimental’ conditions of a rural population living in a highly seasonal environment, our study suggests that brief periods of environmental variation in early life can have long-lasting intergenerational consequences, even under conditions of (relative) affluence in which child survival is high. One major difference between this study and that of Alwasel and colleagues [22,23], which addressed similar questions by examining effects of maternal *in utero* exposure to Ramadan fasting, is our use of cyclical Fourier terms, which (i) allow effects of early birth environment to be examined [23,37,42] without the risk of biologically relevant effects being rejected when trimester-specific cohorts comprise small numbers of individuals [24,48] and (ii) avoids assumptions about the timing of the environmental influence.

As with all non-experimental long-term studies of this kind, the potential for attrition or selection bias needs to be considered. Birth season effects might become apparent because of relaxed selection on phenotypic characteristics associated with conception, birth or survival, causing maternal cohorts, and their offspring, to be comprised of individuals who are more variable in anthropometry than previous generations. However, this would not account for the reversal of the birth season effects. Furthermore, it should be mentioned that among the three cohorts, the proportions of mothers born between July and December (exposed to the hungry season during late gestation) were 49 per cent, 50 per cent and 54 per cent, respectively. In addition, there was no evidence that maternal heterogeneity, as indexed by anthropometric traits, accounted for the patterns among offspring outcome, as might be expected if it were the case that these effects were caused by selection.

The fact that the apparent effects of maternal birth season were not mediated by a mother's own birth/neonatal weight or her adult height indicates that the second generation foetal growth pattern might not be the direct long-term consequence of a first generation growth restriction, as has been found previously [18,25,26]. However, a limitation of this study was that other measures of maternal size that are good candidates for linking maternal early environment with offspring growth, such as measures of pelvic size [25,49,50], were not available. There are several alternative plausible mechanisms by which such intergenerational effects could arise. Foetal growth may be influenced by several maternal physiological factors, such as blood pressure [25,51], glucose–insulin metabolism [27,28,52] and even the hypothalamic–pituitary–adrenal axis [29,53]. These traits may be ‘programmed’ during maternal foetal life, specifically by macronutrients [28,54–56], micronutrients [29,57] or (grand) maternal stress [32–34,58,59], and may thus provide a route for maternal birth season to influence offspring foetal growth. However, in this population, a study of men and women born before 1975 found no effect of birth season on glucose–insulin metabolism [60], but it remains to be studied whether such effects emerge for the later cohorts.

Given that foetal growth is a complex trait determined by both maternal and foetal characteristic, the effects of maternal birth season described here could be either due to epigenetic programming of maternal somatic or gametic cells, or both. If the results observed in this study are owing to environmental effects on methylation, then the window of exposure that is involved in first generation effects on foetal growth is likely different from that which is relevant for second-generation effects. This is because while any first generation effects are probably owing to constrained third trimester growth during the hungry season [35,37], methylation effects probably occur during the first days of embryonic life. Accordingly, in the context of this putative mechanism, the window of interest would be the periconceptual period. The fact that effects of maternal, but not paternal, season of birth were observed, could be considered less consistent with an epigenetic mechanism than one linked to the maternal intrauterine environment.

Interpretation of the inversion of the pattern of the effects of maternal birth season presents another challenge. It is possible that the explanation lies in a combination of different environmental factors. Rates of infection (especially malaria) [38], exposure to aflatoxins [61,62] and pesticide exposure [63] are other external environmental stressors that could have long-term effects whose seasonal patterns may have changed over time, and which could plausibly influence maternal early development. One intriguing possibility is dietary changes owing to supplementation during pregnancy and lactation designed to improve maternal and neonatal outcomes [64–67]. For example, between 1980 and 1984, pregnant women in Keneba were given a daily balanced dietary supplement, which was increased during the hungry season [66,68]. Implicating the effects of supplementation trials in these results might accord well with the abrupt changes observed between cohorts. However, it should be noted that supplementation of pregnant women began once pregnancies were diagnosed, and so supplementation is unlikely to have influenced methylation status, which is likely to be determined in early embryonic life. Therefore, if the results described are owing to an interaction between one or more supplementation trial and maternal birth season, the mechanism would not involve changes to methylation status, although they could involve changes in other maternal traits.

To conclude, our results support the idea that brief periods of early environmental variation in mothers can affect their offspring *in utero* growth, with potentially long-term consequences even in rural populations far from starvation. The unexpected emergence of these intergenerational effects of birth season in recent years suggests that early environmental effects can be apparent despite, or perhaps because of, substantial reduction in child mortality. The persistence of these relationships when controlling for measures of maternal size indicate that the mechanisms behind intergenerational effects of maternal early conditions may not be found in a simple model of maternal physical or physiological constraint and point to the need to account for other aspects of maternal phenotype associated with offspring growth such as pelvic dimensions, physiological parameters like insulin metabolism, glucocorticoid levels, hypertension or epigenetic inheritance [2,3,25,26].
